# Research progress of serum eosinophil in chronic obstructive pulmonary disease and asthma

**DOI:** 10.1515/biol-2022-0779

**Published:** 2023-12-07

**Authors:** Congli Wu

**Affiliations:** Department of Respiratory Medicine, The First People’s Hospital of Lianyungang, Lianyungang City, 222000, China

**Keywords:** eosinophil, chronic obstructive pulmonary disease, asthma, pathogenesis, diagnosis, prognosis

## Abstract

Chronic obstructive pulmonary disease (COPD) and asthma are common airway diseases, and there are similarities and differences between them. Serum eosinophil (EOS) has potential application value in the diagnosis, treatment, and prognosis of COPD and asthma. However, the clinical application value of serum EOS in these two diseases is controversial. In this work, recent research progress on the application of serum EOS in COPD and asthma was analyzed, and the potential application of serum EOS in the two diseases was expounded from several aspects, including the correlation between the pathogenesis of COPD and asthma and EOS, as well as the correlation between the diagnosis, treatment, and prognosis of the two diseases and serum EOS. The results revealed that serum EOS was important in the pathogenesis, diagnosis, treatment, and prognosis of COPD and asthma and exhibited a potential clinical application value. However, further study was needed to evaluate the threshold, which provided guidance for the clinical diagnosis and treatment of COPD and asthma.

Asthma and chronic obstructive pulmonary disease (COPD) are two common airway diseases. COPD is a chronic respiratory disease characterized by persistent respiratory symptoms and airflow limitation, often caused by airway and/or alveolar abnormalities caused by toxic particles or gases [1]. Asthma is a chronic airway inflammatory disease, which involves a variety of cells and cell components. Asthma and COPD are effectively distinguished due to differences in bronchodilation tests [2]. However, patients with both diseases have clinical symptoms such as cough, phlegm, wheezing, and shortness of breath [3]. Some patients may be characterized by asthma–COPD overlapping (ACO), and about 20% of COPD patients are misdiagnosed as ACO [4]. Some researchers have pointed out that chronic airway diseases are the result of the combined action of genes and environmental factors and believe that asthma and COPD are two different manifestations of the same disease [5]. Approximately one-third of patients with COPD have a history of asthma disease, and over 40% of patients with airflow limitation have ACO [6]. Eosinophil (EOS) is a common indicator to evaluate the level of airway EOS inflammation, which can guide the selection of stable drugs in COPD patients [7]. The main characteristic of asthma is eosinophilic airway inflammation. Serum EOS is a commonly used marker of eosinophilic airway inflammation, which can be used as a predictor of asthma severity and prognosis [8]. However, the value of serum EOS in the exacerbation risk and treatment guidance of COPD and asthma is still controversial, and currently, there is no serum EOS threshold for the diagnosis of COPD and asthma [9]. Therefore, this work reviewed the research progress of serum EOS in COPD and asthma, so as to offer some reference for the value of serum EOS in diagnosing and treating COPD and asthma.

## 1 Physiological and pathological characteristics of EOS

**Figure 1:**
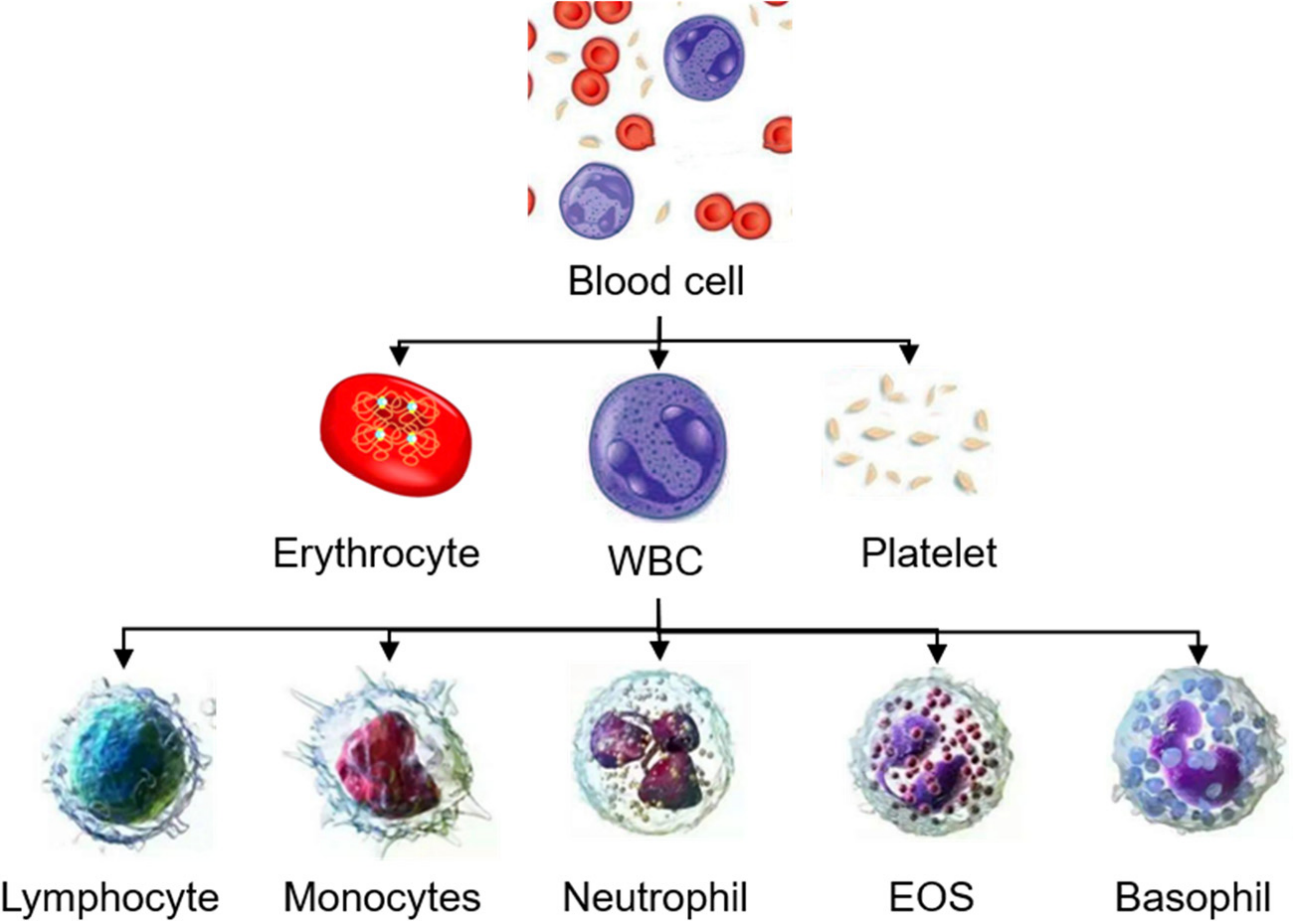
Distribution of EOS in blood cells [17].

EOS is a kind of innate multifunctional immune cells, and the number of EOS in tissues is 100 times that in peripheral blood [10]. As an immune leukocyte, EOS accounts for less than 5% of total leukocytes (Figure 1). EOS is mainly formed in the bone marrow and enters tissues after staying in blood for about 25 h, but its duration in different tissues is uncertain [11]. Under normal circumstances, EOS is mainly distributed in the gastrointestinal tract and the thymus. When the body appears infection and other pathological states, a large amount of EOS will migrate to the lungs, promoting the abnormal release of inflammatory factors in the lung airway, resulting in inflammatory response. EOS can express immunoglobulin IgG, IgA, complement receptor, cytokine receptor, leukotriene (LT) receptor, prostate receptor, toll-like receptor, and other cell surface receptors [12,13,14]. The binding of these receptors to the embryo can stimulate the change of EOS activity and release lipid mediators and reactive oxygen species. The cell products of EOS [15,16] mainly include (1) a variety of positive particle proteins, basic proteins, and other granular media; (2) lipid mediators such as LT C4/FGE2/thrombus and platelet-activating factor (PAF); and (3) cytokines and chemokines of transforming growth factor β and interleukin (IL)-4, IL-5, IL-8, IL-10, and IL-12. The above EOS cell products participate in the occurrence and development of diseases by inhibiting airway cilia function, stimulating bronchoconstriction, promoting airway remodeling, and immune response.

**Figure 2:**
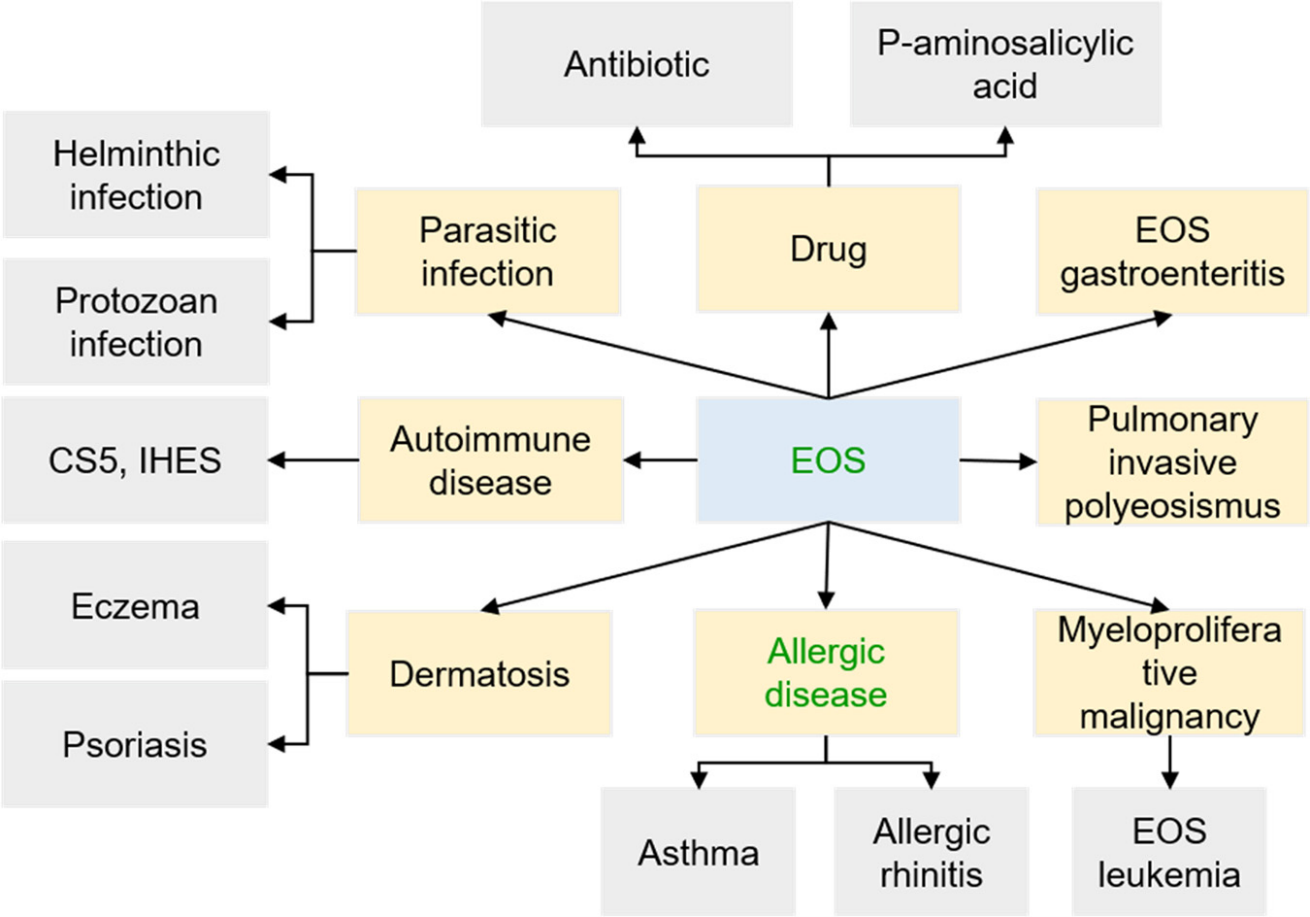
Influencing factors of EOS level change.

Serum EOS is derived from bone marrow hematopoietic stem cells and has good short-term stability, which is affected by age, sex, baseline EOS level, and drug use [18]. Studies have shown that serum EOS levels in adults do not change with age but are susceptible to factors such as asthma, smoking, COPD, metabolic syndrome, and obesity, thus affecting serum EOS stability [19]. Serum EOS levels are associated with serum infections, dermatosis, allergic diseases, autoimmune diseases, myeloproliferative malignancies, pulmonary invasive hyper-EOS and EOS gastroenteritis [20], and serum EOS levels are also altered by simultaneous administration of drugs [21]. The influencing factors of EOS level change are shown in Figure 2. Serum EOS is highly variable [22], and its value in the diagnosis and treatment of respiratory diseases and clinical decision guidance remains to be further verified. EOS has a variety of functions, including cell synthesis and release of active substances, removal of harmful substances, tissue damage and repair, immune regulation, and other processes, as well as inhibiting tumor cell growth and promoting tumor cell apoptosis [23]. EOS mainly plays a role in host immune defense and post-parasitic immunity in allergic diseases, and there is a certain correlation between the amount of EOS and the clearance rate of viruses and pathogenic bacteria, suggesting that EOS has antibacterial properties [24]. In the lung, asthma is characterized by allergic reactions and Th2 lymphocyte-mediated eosinophilic inflammation. Acute exacerbation of COPD (AECOPD) exacerbation refers to exacerbation of airway inflammation in some AECOPD patients [25].

## 2 Research progress of serum EOS in COPD

### 2.1 Overview of COPD

**Figure 3:**
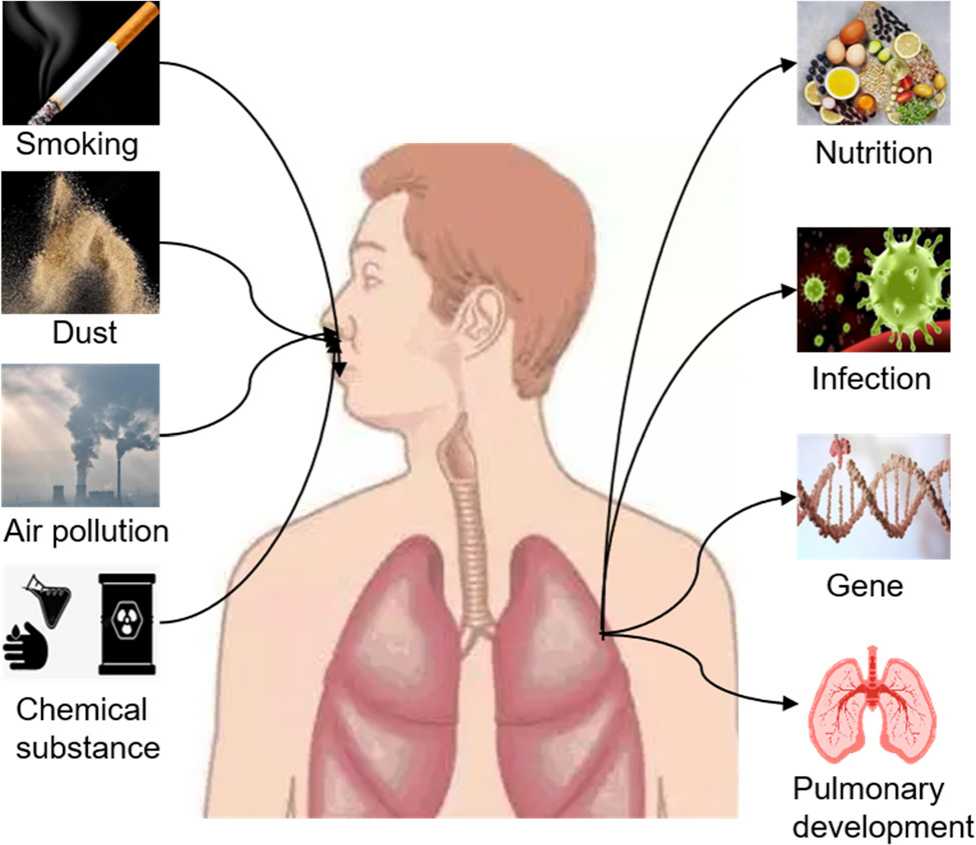
Risk factors of COPD.

COPD has become one of the common and frequently occurring diseases worldwide and is the fourth leading cause of human death worldwide. Relevant statistics show that in 2015, about 3.2 million people died due to COPD worldwide, an increase of 11.6% over 1990 [26]. The case fatality rate of COPD is increasing year by year and is expected to become the third leading cause of death in the world by 2030. China, as an agricultural country and a large population, currently has about 100 million people suffering from COPD. The prevalence rate of COPD in people over 40 years old is as high as 13.7%, and the incidence rate of COPD in rural people over 60 years old is more than 27% [1]. The main clinical manifestations of COPD are intermittent cough, sputum, shortness of breath or dyspnea, wheezing, chest tightness, chest congestion with loss of appetite, peripheral muscle atrophy and dysfunction, mental depression, and anxiety [27]. Current research results show that the occurrence of COPD is mainly influenced by genetic factors and environmental factors, and the genetic factors are mainly manifested in antitrypsin loss, and the proportion of COPD caused by antitrypsin loss is about 1.5% [28]. Environmental factors mainly include living area, altitude, age, smoking, dust exposure occupation, biofuel exposure, atmospheric smog, and respiratory tract infection (Figure 3). The incidence of COPD was positively correlated with age and inversely correlated with altitude. In stable COPD patients, under the stimulation of pathogen infection, improper diet, inhalation of allergens, and other inducing factors, the disease will be further aggravated and transformed into AECOPD. COPD can cause a series of complications, which not only affect lung function but also involve other organs outside the lung [29]. Therefore, timely and accurate diagnosis and evaluation of COPD and its severity have important guiding significance for the evaluation and treatment of COPD progression.

### 2.2 Pathogenesis of COPD and its relationship with EOS

**Figure 4:**
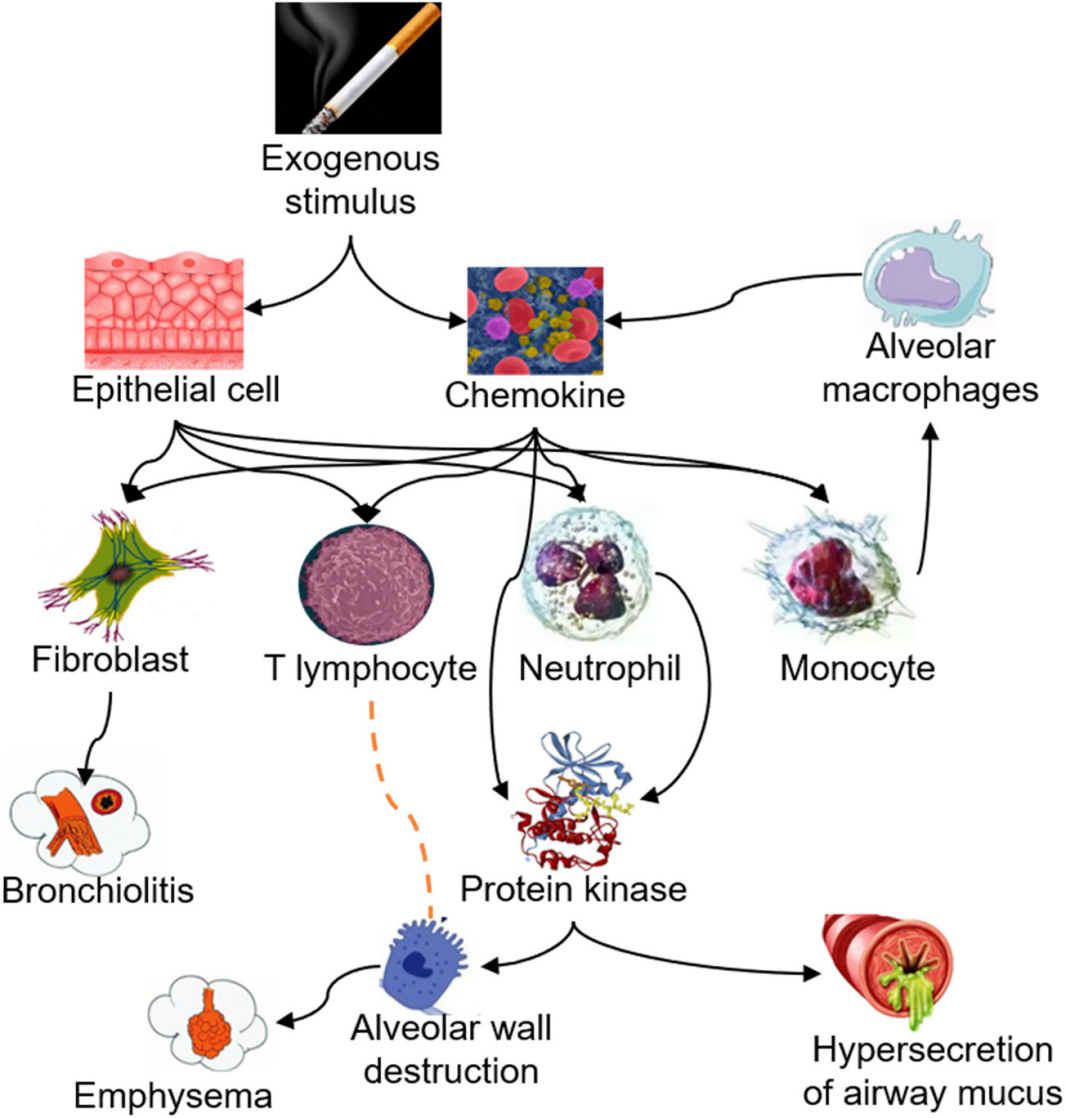
Pathogenesis of COPD [34].

Pathogenesis of COPD is still unclear. However, it is widely believed that the pathogenesis of COPD is related to airway inflammation, immune mechanism, oxidative stress imbalance, protease/antiprotease imbalance, emphysema overinflation, and inflation [30]. The specific pathogenesis of COPD is illustrated in Figure 4. At present, a large number of studies have pointed out that COPD is a chronic inflammatory development, in which a large number of neutrophils (NEU), macrophages, and T cells are involved, and its occurrence is correlated with the release of IL-8, LT B4, and other factors [31,32]. Studies have pointed out that stimulation of external factors (such as smoking) will cause B cells in the body to secrete a large amount of IgIC, which will aggravate the inflammatory response of the body [33]. On the other hand, CD8+ lymphocytes play an important role in the development of COPD. In terms of oxidative stress, inhaled particulate matter can produce oxidative free radicals, which can reduce the endogenous antioxidant products of the body, promote the oxidative stress reaction of the body, and aggravate the deterioration of lung function. The imbalance between excessive lipid peroxidation and antioxidant is closely related to COPD. In terms of protease/antiprotease imbalance, the content of protease in COPD patients is significantly increased, the increased protease causes pulmonary inflammatory infiltration, and fibroblasts produce a large number of factors such as NE and MMP, which further promotes the occurrence of emphysema, aggravates the inflammatory response, and promotes the severity of COPD. Lack of antiprotease will cause lung protease hydrolytic injury, resulting in chronic cell injury.

**Figure 5:**
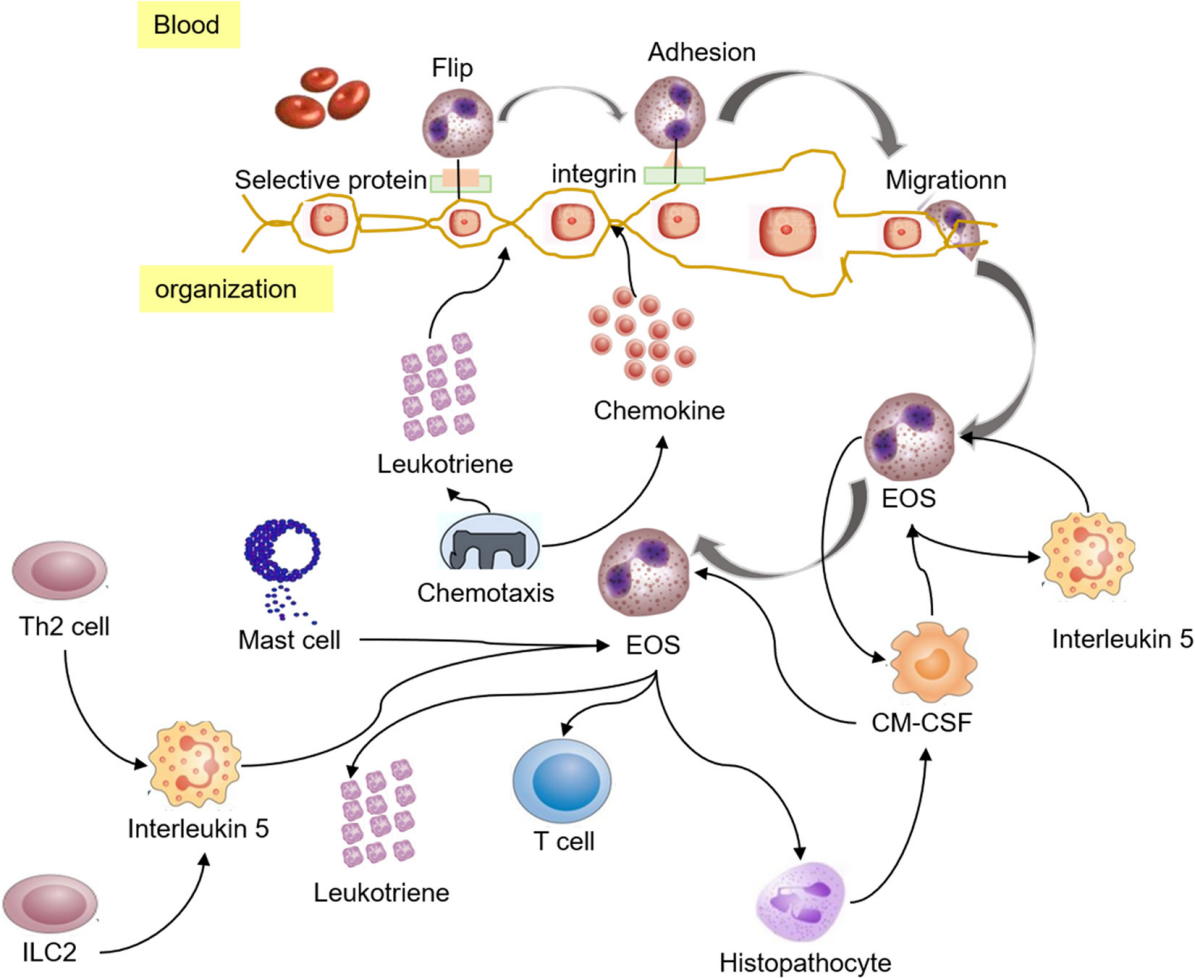
Differentiation and maturation of EOS [37].

COPD exhibits obvious clinical heterogeneity. Most patients present with NEU inflammation, but some patients still present with increased EOS count. NEU are the main inflammatory cells of AECOPD, and bacterial infection can cause elevated NEU levels in the respiratory tract. At the same time, studies have found that EOS plays an important role in inflammation in 10–40% of COPD patients [35], suggesting that EOS may have a certain correlation with the occurrence and development of COPD. The differentiation and maturation process of EOS in the respiratory system is demonstrated in Figure 5. Stimulation of external factors (smoking, harmful substances) and viral infection can cause respiratory epithelial cells to release a large amount of thymic stromal lymphopoietin and IL-33, promote the change of IL-5 secretion, degranulate EOS, promote tissue damage, and aggravate inflammation. IL-5 is one of the main regulatory factors of EOS and is related to EOS production, release, migration, and survival. The level of IL-25 and IL-33 in lung tissue can affect the level of congenital non-T cells and thus regulate the up-regulation of IL-5 level and promote the increase of EOS level. Meanwhile, studies have shown that IL-33 can enhance Th2 cells, improve the survival rate of EOS, and induce superoxide anion production and degranulation of EOS [36]. Exogenous molecules such as nitric oxide and ozone would increase the expression level of EOS chemokines in airway epithelial cells, so that peripheral serum EOS could play a role in vascular endothelial cell adhesion and chemotaxis.

**Figure 6:**
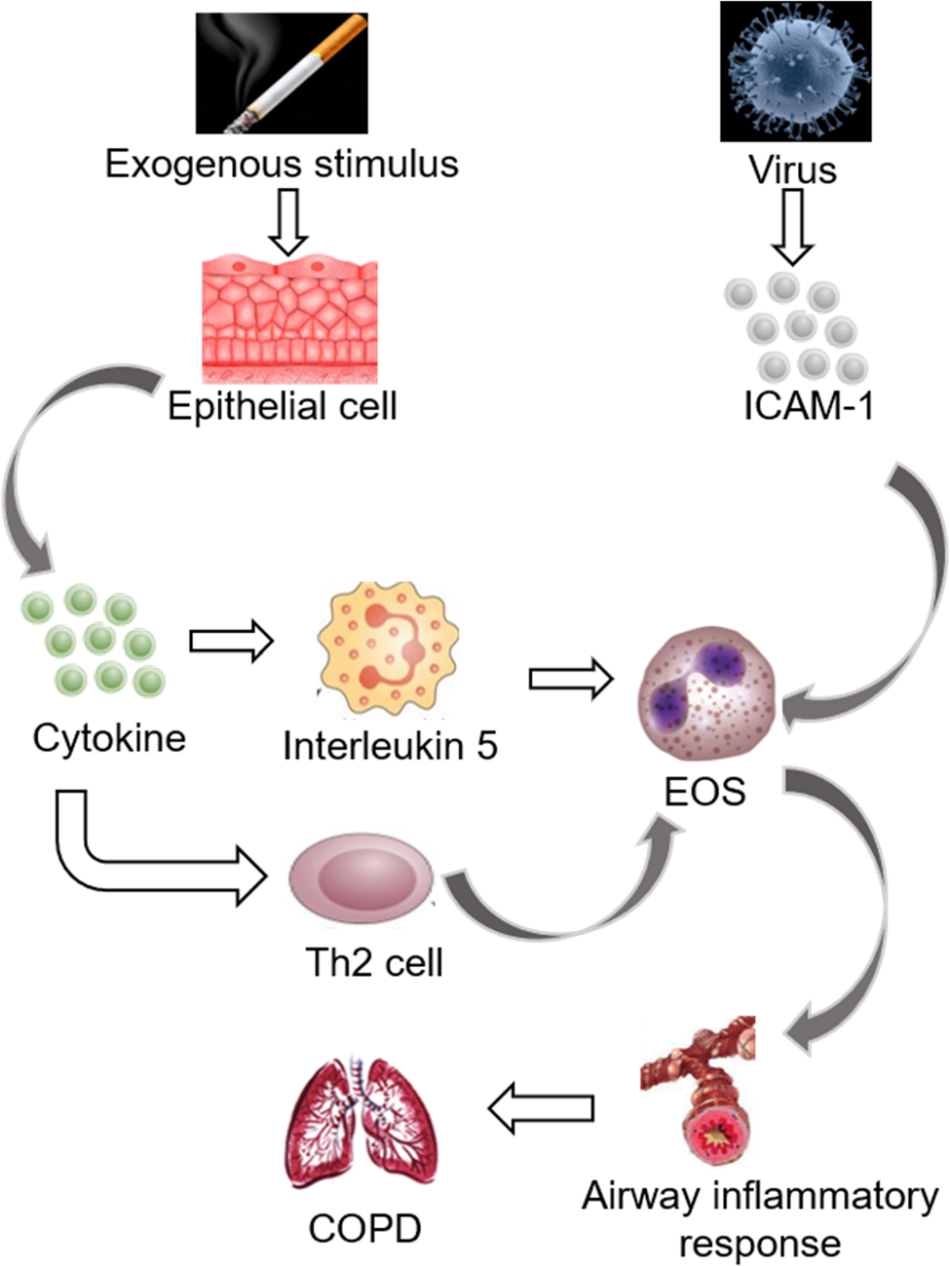
The incidence of COPD and its relationship with EOS.

EOS participates in Th2 type immune response. Under the action of cytokines, EOS migrates to inflammatory tissues and releases a large number of cytokines, lipid mediators, and granulocins, among which granulocins are harmful to bronchial epithelium and closely related to airway inflammation [38]. In addition, activated EOS will release PAF, LTs, and other inflammatory mediators, causing bronchial contraction and mucosal microvascular leakage, and aggravating tissue damage. Bacterial or viral infections cause secretory IgA levels to rise, which activates EOS and releases eosinophilic positive ions and other proteins into body fluids, which increases airway mucus secretion, reduces ciliary movement, and leads to persistent airflow limitation in patients [39]. At the same time, it has been shown that the level of intercellular adhesion moleculin-1 increases during viral infection, which promotes the migration of EOS in the peripheral blood to the airway and aggravates airway inflammation [40,41]. Figure 6 demonstrates the incidence of COPD and its relationship with EOS.

### 2.3 Correlation between COPD clinical features and EOS

The subjects have been grouped based on serum EOS concentration differences [42,43]. Serum EOS levels greater than 200/μL or 2% significantly increased sputum EOS count and IgE, with obvious differences in age, sex, obesity, and smoking indices, and increased wheezing symptoms [44]. In addition, the score of Saint George’s respiratory questionnaire was increased, and the values of lung function indexes forced expiratory volume in 1 second (FEV1), forced vital capacity (FVC), and FEV1/FVC were decreased remarkably. Serum EOS levels were negatively correlated with biochemical indices such as white blood cell, NEU%, and c-reactive protein. Patients with AECOPD with EOS levels below 2% had poorer lung function and were at higher risk for pneumonia, respiratory failure, and chronic pulmonary heart disease. At the same time, it has been reported that [45] clinical characteristics and lung function indices showed no great differences when serum EOS level was greater than 300/μL. Therefore, the correlation between the clinical characteristics of COPD and serum EOS remains controversial.

### 2.4 The relationship between diagnosis and treatment of COPD and EOS

There are few studies on the direct correlation between COPD diagnosis and EOS levels. In the *Global Initiative for Chronic Obstructive Lung Disease* scale, emphysema and NEU inflammation were more common in patients with grades B and D COPD. Chronic bronchitis is more common in patients with grades A and C COPD [8]. Induced sputum cell counting is the gold standard for evaluating airway EOS inflammation, but it is easy to be affected by subjective factors, difficult to take samples, and has certain limitations in clinical application. Serum EOS count is correlated with induced sputum cell count, but its correlation with COPD remains controversial. Some studies showed that serum EOS was correlated with induced sputum EOS, but the correlation was weak. The false positive rate of sputum EOS predicted by serum EOS was as high as 72.00% [46]. Ortega et al. [47] pointed out that serum EOS levels were significantly correlated with sputum EOS levels when serum EOS levels were lower than 210.6/μL but were susceptible to factors such as age and cardiovascular complications. Fractional exhaled nitric oxide (FENO) is a marker of inflammation in airway EOS with induced sputum cell count as the gold standard. Serum EOS and FENO levels both predicted the level of airway EOS inflammation, and serum EOS was superior, but serum EOS combined with FENO did not improve the diagnostic accuracy [48]. Serum EOS levels in COPD were monitored for 7 years [49] and showed great variability throughout the course of the disease. However, Negewo et al. [50] pointed out that serum EOS levels in COPD patients could be reasonably repeatable within 1 year. Therefore, the application value of serum EOS in the diagnosis of COPD needs further investigation.

The main inflammation was EOS. Currently, according to the different exacerbations of COPD, COPD is divided into mainly bacterial, mainly viral, mainly EOS, and a certain type of “oligo-immune” inflammation. According to different types of COPD diagnosis, the choice of treatment plan for patients can be guided. For COPD patients mainly with NEU, antibiotics can be used, and for COPD patients mainly with EOS, glucocorticoid can be used. Both clinical studies and Global Initiative for Chronic Obstructive Lung Disease concluded that serum EOS levels could guide the selection of glucocorticoid therapy in stable COPD. Studies have pointed out [51] that the mechanism of glucocorticoid in treating COPD, which is dominated by EOS, is mainly related to the regulation of EOS expression level by hormones. Hormones bind to the receptors on the nucleus and are transported to the nucleus to inhibit the synthesis of pro-inflammatory proteins and promote the apoptosis of EOS and other inflammatory cells, so as to achieve the purpose of treating COPD. An investigator [52] who administered hormone therapy to patients with COPD based on EOS found that lung function improved and hormone use was reduced by 49% after hormone therapy. Therefore, serum EOS is a potential biomarker for evaluating the efficacy of AECOPD. COPD patients with elevated serum EOS levels [53] responded to betamethasone/formoterol therapy. Patients with moderate-to-severe COPD treated with fluticasone/vilanterol had a lower incidence of AECOPD when serum EOS level was greater than 2%. There were also studies [54] that there was a bias in determining the treatment of COPD patients based on serum EOS levels.

### 2.5 Relationship between prognosis of COPD and EOS

The relationship between COPD prognosis and EOS mainly includes the following aspects. (1) Serum EOS reduction may be a marker of poor prognosis in COPD and was linked with increased mortality and length of hospital stay in patients with AECOPD. In addition, serum EOS reduction was also obviously linked with the duration of hospital stay, the need for mechanical ventilation, in-hospital death, and 30-day readmission and mortality in COPD patients. (2) Serum EOS increase was an independent predictor of AECOPD combined with pneumonia mortality. A retrospective analysis [55] of the correlation between serum EOS levels and length of ICU stay and mortality in COPD patients found that patients with serum EOS levels greater than 2% had significantly lower length of ICU stay and mortality than patients with serum EOS levels less than 2%. Barnes et al. [56] found that patients with AECOPD serum EOS ≥2% had lower CRP levels and significantly shortened hospital stay, suggesting that serum EOS level could be used as a biomarker for prognosis of COPD. Watz et al. [57] found that patients with serum EOS level greater than 2% had much shorter hospital stays than patients with serum EOS level less than 2% during the 1-year observation period, but there was no statistical difference in the rate of re-hospitalization and the time to first exacerbation. At the same time, there were no significant differences in respiratory symptoms, pulmonary function, and exacerbation rate in COPD patients with different serum EOS levels [58], suggesting that serum EOS levels could not be used as biomarkers of COPD phenotype. Therefore, the value of serum EOS levels in the prognosis of COPD needs further investigation.

## 3 Research progress of serum EOS in asthma

### 3.1 Overview of asthma

Asthma is a common and frequent disease in the respiratory department. With the aggravation of global air pollution, asthma incidence is increasing year by year in the time range, which is a global problem threatening human health. There are about 300 million asthma patients worldwide, and the incidence rate is 1–18% [59]. It is expected that the number of asthma patients worldwide will exceed 300 million by 2025. The asthma mortality rate is between 1.6 and 36.7 per 100,000, with the highest incidence among children. The asthma incidence of children aged 13–14 years is 0–30% [60]. There are about 30 million asthma patients in our country, accounting for 1/10 of the world’s asthma patients. The domestic asthma incidence is 0.5–5%, and the incidence of asthma in children aged 13–14 is 3–5% [61]. China has become one of the countries with a high asthma case fatality rate due to the factors of long-term poor control and untimely treatment. Therefore, it is of positive significance for human health to develop asthma prevention and control actively.

**Figure 7:**
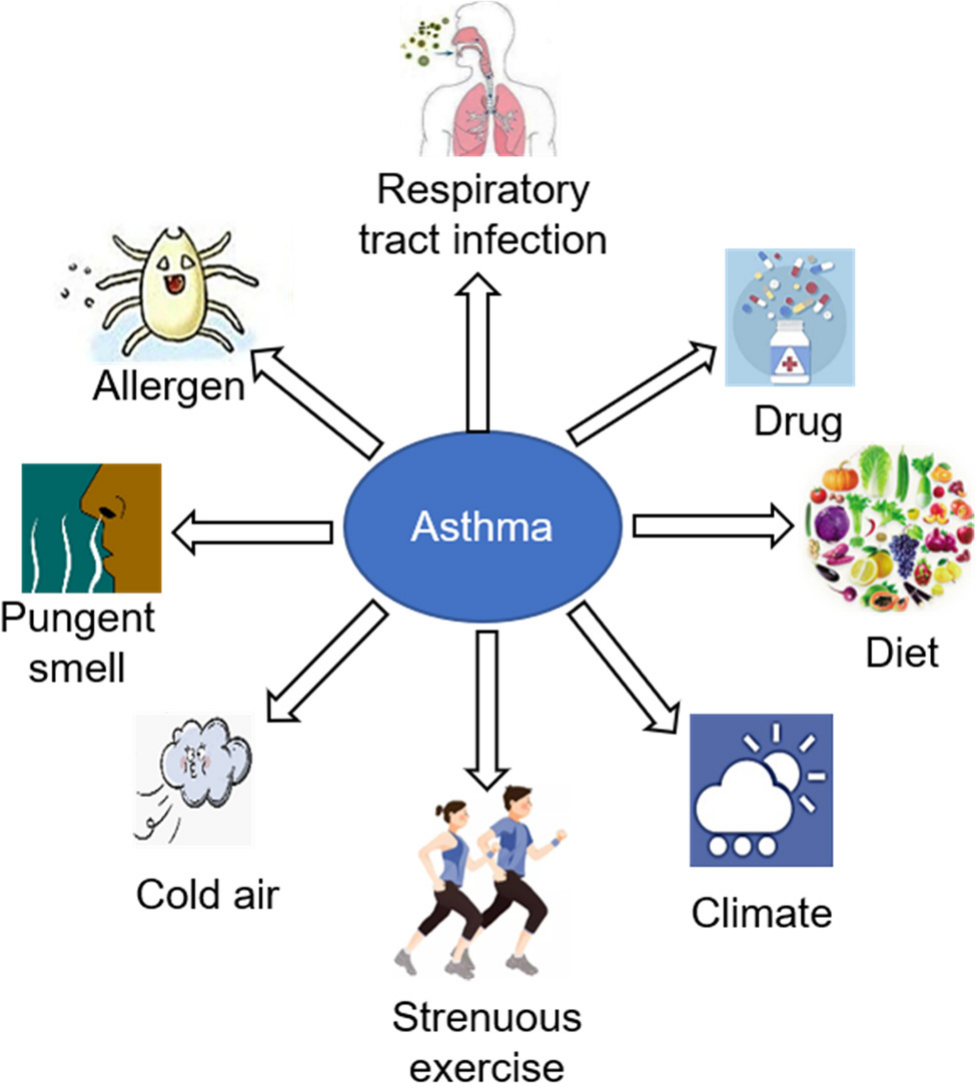
Environmental factors of asthma onset.

Asthma is mainly caused by both genetic and environmental factors. According to statistics, approximately 50.9% of asthma patients have a family history of allergies, and 37.5% of patients have a family history of asthma [62], suggesting that the occurrence of asthma may be related to gene regulation and immune regulation. Environmental factors mainly include external inhalants, infection, diet, climate, drugs, etc., external inhalants specific allergens (such as pollen, dust mites, fungi, and animal dander), and non-specific irritants (such as sulfuric acid, sulfur dioxide, formaldehyde, and formic acid) (Figure 7). After external inhalants enter the body, the airway mucosa will accumulate a large amount of external inhalants, promoting the occurrence of local or systemic immune response, and then leading to the occurrence of airway allergic inflammation [63]. When the body is infected with bacteria, viruses, mycoplasma, and other infectious sources will cause damage to the respiratory epithelium and promote the increase of respiratory responsiveness [64]. Long-term repeated infection is closely related to asthma occurrence. Diet is one of the factors that lead to asthma development in infants. Temperature, air pressure, humidity, strenuous exercise, drug use, and other factors are correlated with asthma development [65].

Asthma is a chronic airway inflammatory disease involving a variety of cells and cell components, which is clinically manifested by repeated wheezing, shortness of breath, chest tightness, cough, etc., which gets worse at night or early morning, most patients can be relieved by themselves or be relieved by treatment [66]. COPD patients have obvious airflow limitation, and their clinical symptoms are similar with little difference to asthma [67,68]. The differences in clinical and physiological characteristics between asthma and COPD are listed in Table 1.

**Table 1:** Clinical and physiological characteristics between asthma and COPD

	COPD	Asthma
Airway hyperreactivity	Reactive with relatively lower responsiveness, less susceptible to stimuli	Increased reactivity, easily triggered, leading to acute attacks
Etiology	Factors such as smoking and prolonged exposure to air pollutants	Caused by allergens, airway inflammation, and airway hyperresponsiveness
Airway inflammatory properties	Chronic inflammation caused by NEU and macrophages	Increased EOSs, inflammation mediated by T lymphocytes
Major inflammatory cell	NEU	EOS
Major lesion site	Airway wall mucosal layer and deep tissue	Airway wall mucosal layer
Airflow limiting mechanism	Irreversible airway inflammation and structural damage	Reversible airway spasms and increased airway mucus secretion
Airway obstruction	Progressive aggravation, irreversible	Changeable and reversible
Cell types of sputum	Increased EOSs	Increased EOSs
Cytokine	IL-1β, IL-6, IL-8, and TNF-α	IL-4, IL-5, IL-9, and IL-13
Inflammatory cell	NEU and macrophages	T lymphocytes and EOSs
Bronchiolar mucus cells	Tissue metaplasia and hyperplasia	Epithelial exfoliation
Pathological features	Parenchymal cell destruction, emphysema, and massive mucus	Parenchymal cells intact, no emphysema, and exudative mucus

### 3.2 The mechanism of asthma and its relationship with EOS

**Figure 8:**
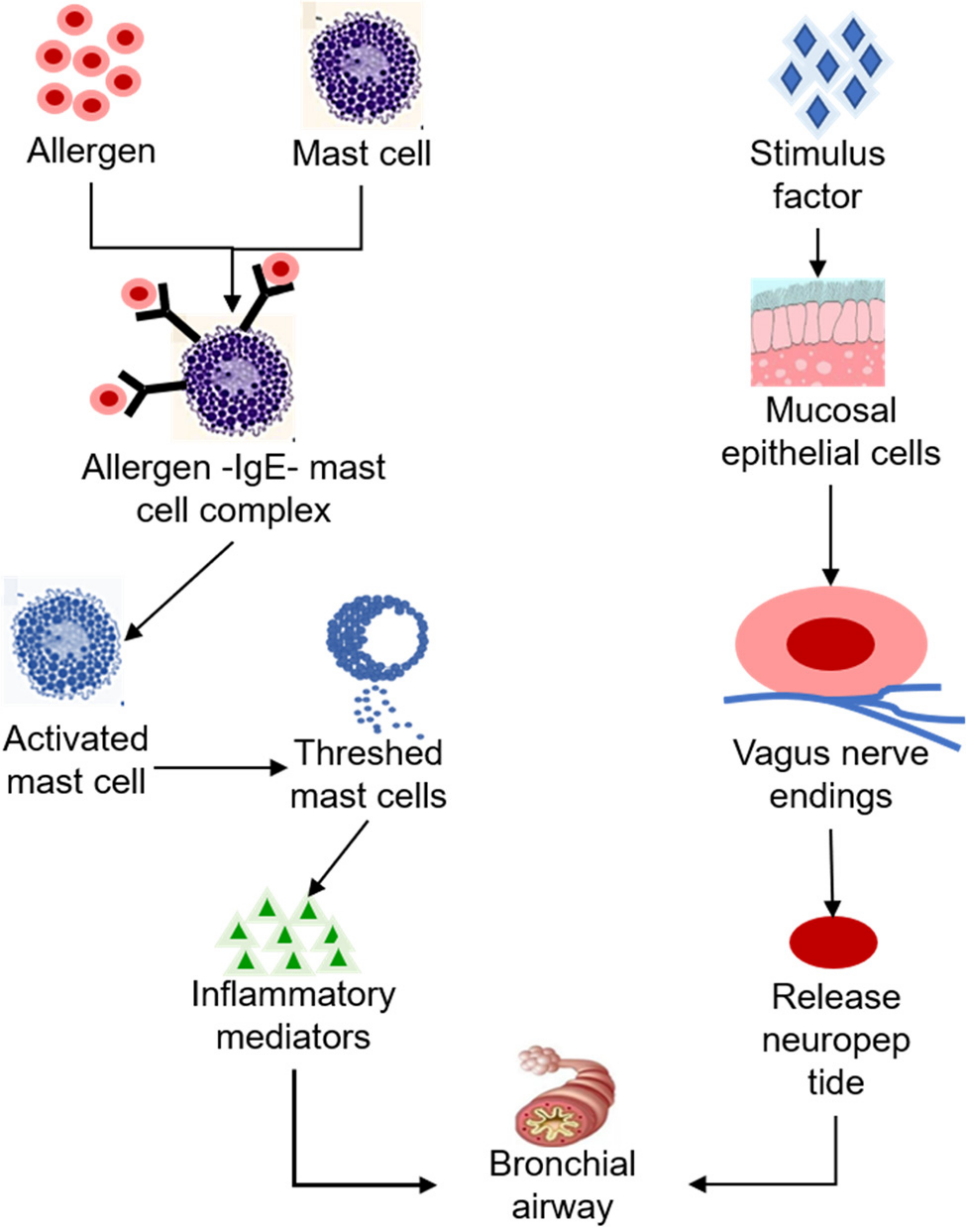
Mechanism of asthma occurrence.

Asthma is a chronic inflammatory disease of the airway involving EOSs, mast cells, Th2, and other inflammatory cells, airway epithelial cells, and cell components. According to the classification and counting of sputum cells induced by airway inflammation indicators, asthma can be divided into eosinophilic, neutrophilic, mixed granulocyte, and oligoinflammatory cell types. Allergens are one of the main factors in the occurrence and development of asthma. The serum IgE level in asthma patients is elevated, which is one of the key links causing the occurrence and development of asthma. Chronic airway inflammation is the basis of asthma. The mechanism of asthma development mainly includes the following aspects [69–72]. (1) Allergy: after exposure to allergens, macrophages, lymphocytes, and granulocytes form antigen-presenting effect, which activates T lymphocytes and secretes a large amount of IL factors. Furthermore, B lymphocytes are regulated to increase IgE levels and adhere to mast cells, EOS, and basophil granulocytes, leading to chronic inflammatory pathological reactions in the body. When the body is exposed to allergens again, the release of inflammatory factors increases, promoting the occurrence of bronchial mucosal inflammation. (2) Airway inflammation: it is generally believed in current studies that asthma is related to the imbalance of Th1/Th2 ratio in the body. When the level of Th2 in the body is high, the expression of cytokines such as IL-4, IL-5, IL-6, and IL-13 will increase obviously. IL-4 can regulate IgE expression level and promote IgE-mediated humoral lymphatic response, resulting in asthma. IL-5 is mainly produced by mast cells, EOS, and TH2 cells. Massive secretion of IL-5 promotes the proliferation of EOS and migrates into the respiratory tract, causing airway epithelial cell damage and airway hyperresponse. The synergistic action of IL-4, IL-5, and IL-6 can stimulate the change of IgA secretion and mediate delayed asthma reaction. IL-13 may promote the synthesis of immunoglobulin IgE, which causes EOS to adhere to airway epithelial cells and trigger an inflammatory response. In addition, IL-13 promotes mucus secretion and participates in airway hyperresponsiveness. Some studies have shown that [73] the blood IL-10 content of asthma patients is significantly reduced, suggesting that IL-10 content is correlated with the occurrence and aggravation of airway inflammation. TNF-α is involved in the migration, adhesion, and infiltration of inflammatory cells, which is related to the occurrence of airway inflammation. (3) Airway hyperresponsiveness: when the airway is stimulated by sensitizing sources, it will cause chronic inflammation of the bronchial mucosa, expose the infected nerve endings of smooth muscle, cause premature and over-strong contraction and spasm of the airway, and eventually lead to asthma. (4) Airway remodeling: airway remodeling is the main pathological feature of asthma, which is mainly manifested by the damage and abnormal proliferation of airway smooth muscle cells. (5) Neuroregulatory mechanism: sensitizer stimulation can cause airway sensory nerve fibers to release peptide substance P, promote airway contraction, lead to airway mucus overreaction, cause inflammation in the body, and eventually lead to asthma. At present, there are few studies on the mechanism of neural regulation, and further studies are needed. (6) Emotional stimulation: studies have pointed out that emotional changes can stimulate cerebral cortex excitement, cause vagus nerve excitation, increase the release of acetylcholine in the body, and increase bronchial smooth muscle tension, leading to conditioned immune regulation. In addition, emotional stimulation will cause changes in the regulation of the endocrine system of the body through the hypothalamic–pituitary–adrenal axis, which will lead to changes in the release of inflammatory factors, increase of mucus secretion, decrease of ventilation ability, and ultimately lead to asthma [74]. The specific mechanism of asthma occurrence is displayed in Figure 8.

**Figure 9:**
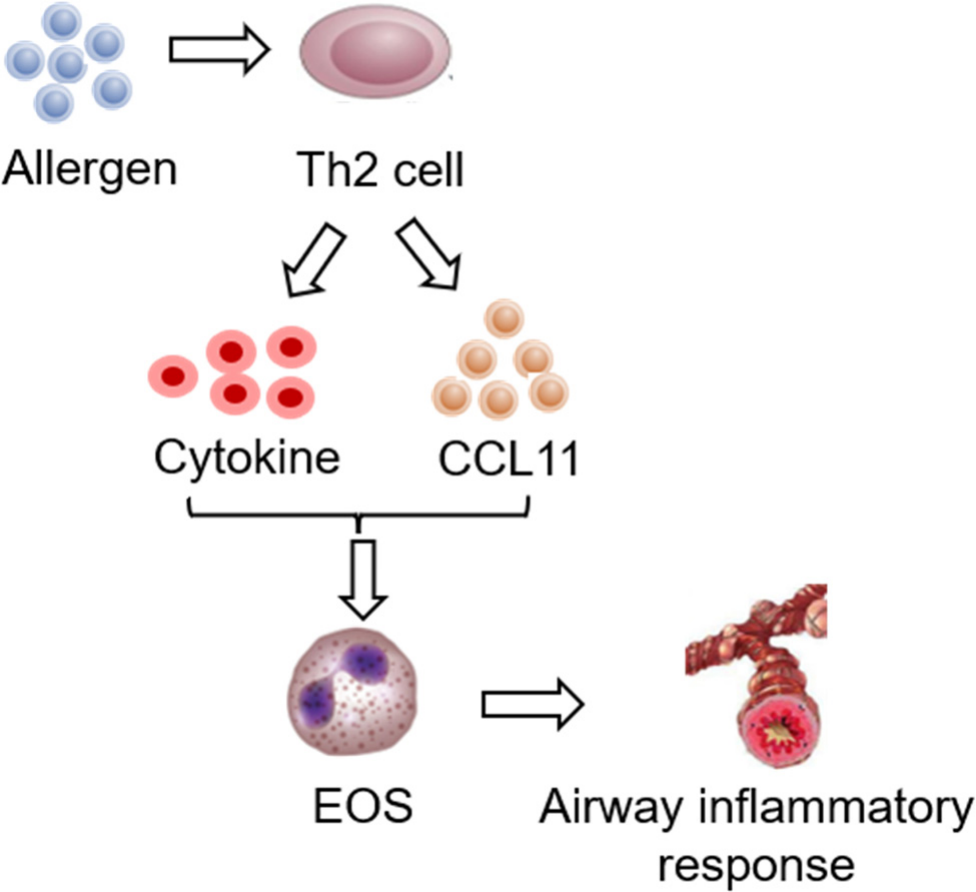
Relationship between asthma occurrence and EOS.

Th2-type immune response plays a key role in asthma development. Stimulation of allergens can increase the release of cytokines such as IL-4, IL-5, and IL-13 in Th2 cells in the body, promote the differentiation, recruitment, and activation of EOS in airway mucosa, further release a large number of inflammatory mediators, promote the occurrence of endogenous eosinophilic inflammatory reaction, and finally, cause asthma. Cytokines secreted by Th2 act together with CCL11 (eotaxin-1) to exert an important role in EOS recycling and allergic inflammation [75]. IL-4 and IL-13 can promote the expression of EOS chemokines, promote EOS adhesion and migration in the airway, increase the amount of EOS in the airway, and then secrete a large amount of granuloprotein, cytokines, and chemokines, leading to the damage of airway endothelial cells and mucous membranes, and eventually trigger asthma [76]. IL-5 is involved in the differentiation, maturation, and activation of EOS. EOS is a major inflammatory effector cell of asthma, involved in airway hyperreactivity, smooth muscle hypertrophy, and airway remodeling [77]. The relationship between asthma occurrence and EOS is illustrated in Figure 9. Studies have found that there is obvious eosinophilic inflammation in the airway sputum and blood of asthma patients in remission, to the extent that the thickness of the basement membrane is positively correlated with the eosinophilic inflammation of the airway wall.

### 3.3 Application of serum EOS in asthma evaluation

Tracheobronchial endoscopy, pulmonary function testing, and respiratory mucosal biopsy are commonly used methods for diagnosing and assessing asthma in clinical practice. Among these, pulmonary function testing might pose challenges for young children or patients with limited cognitive abilities. Asthma attacks are often transient, and if an attack does not coincide with the testing process, the results of pulmonary function testing might not capture the typical pathophysiological characteristics of the patient. Additionally, certain cases of early-stage or mild asthma might not exhibit clear airflow limitation [78], making pulmonary function testing unable to accurately detect these situations. Tracheobronchial endoscopy, being an invasive diagnostic technique, can cause discomfort and mild pain in patients and may lead to complications such as laryngospasm and bleeding [79]. It cannot comprehensively reflect dynamic changes in the airways and cannot provide quantitative data on lung function and airflow limitation. Respiratory mucosal biopsy involves invasive procedures, and therefore, many patients might be resistant to it. Sputum EOS is one of the commonly used methods to evaluate eosinophilic airway inflammation, and there is a significant correlation between sputum EOS and persistent airway inflammation, decreased lung function, and asthma severity [80]. Eosinophilic airway inflammation can be determined by bronchoalveolar lavage and increased EOS in induced sputum analysis. Several studies [81–83] have indicated that serum EOS is significantly correlated with sputum EOS, and serum EOS is a simple, safe, rapid, and potential marker of eosinophilic airway inflammation. A meta-analysis [84] showed that blood EOS level could better reflect the status of airway eosinophilic inflammation. It was found [85] that serum EOS percentage threshold of 2.7% could effectively predict eosinophilic airway inflammation. Castro et al. [86] showed that serum EOS percentage threshold of 3.0% could provide 78 and 91% sensitivity and specificity in predicting eosinophilic airway inflammation, respectively, suggesting that serum EOS could be a good substitute for sputum EOS. However, due to differences in research methods and severity of asthma patients, the optimal threshold value of serum EOS in asthma diagnosis is still controversial and needs to be further studied. FENO refers to the concentration of nitric oxide measured from exhaled gas at the end of expiration. Nitric oxide is primarily produced by airway epithelial cells and EOSs, and its levels are correlated with the degree of airway inflammation. In asthma patients, eosinophilic inflammation is a common type of inflammation, and FENO can serve as one of the representative indicators to assess airway inflammation. By measuring the FENO levels, it is possible to indirectly understand the extent of airway inflammation and conduct an assessment [87]. Elevated FENO levels are typically associated with increased eosinophilic inflammation, suggesting a potentially more severe degree of airway inflammation. Conversely, lower FENO levels may indicate relatively milder airway inflammation or a period of remission [88]. FENO cannot be used as a standalone method for diagnosing asthma, nor can it comprehensively evaluate the overall state of asthma control. It is usually employed in combination with a patient’s symptoms, clinical presentations, pulmonary function tests, and other auxiliary examination results to make comprehensive judgments and assessments.

### 3.4 Application of serum EOS in asthma severity and prognosis

Patients with eosinophilic asthma had lower lung function impairment, symptom control, and quality of life than those with non-eosinophilic asthma, and serum EOS count was significantly correlated with asthma severity. Therefore, some researchers pointed out that the increase in serum EOS count suggests that the decrease in lung function in asthma patients, and timely and effective treatment should be given [89]. Mallah et al. [90] investigated the correlation between serum EOS and lung function in asthma patients and found that serum EOS level was significantly negatively correlated with the value of FEV1, an indicator of lung function. Serum EOS level increased, which promoted airflow obstruction and further promoted lung function decline. Rupani and Teague [91] pointed out that serum EOS level was significantly positively correlated with the risk of asthma attack, and serum EOS count greater than 0.4 × 10^9^/L suggested the progression of asthma attack or poor asthma control. Serum EOS counts [92] were associated with the severity of respiratory failure in acute asthma attacks, suggesting that serum EOS counts could be used to predict the risk of asthma deterioration when the EOS count was greater than 1.2 × 10^9^/L. Simultaneous asthma patients may have a severe eosinophilic phenotype. These results suggest that serum EOS may be a potential asthma predictor biomarker.

## 4 Summary

This work examined the potential application value of serum EOS in patients with COPD and asthma pathogenesis, as well as the correlation between these two diseases. Serum EOS plays an important role in the pathogenesis, diagnosis, treatment, and prognosis of COPD and asthma and has potential clinical application. However, the assessment threshold still needs further study, which provides guidance for treating COPD and asthma in clinic.
